# Innovative Therapeutic Approaches in Non-Alcoholic Fatty Liver Disease: When Knowing Your Patient Is Key

**DOI:** 10.3390/ijms241310718

**Published:** 2023-06-27

**Authors:** Marta Alonso-Peña, Maria Del Barrio, Ana Peleteiro-Vigil, Carolina Jimenez-Gonzalez, Alvaro Santos-Laso, Maria Teresa Arias-Loste, Paula Iruzubieta, Javier Crespo

**Affiliations:** 1 Gastroenterology and Hepatology Department, Clinical and Translational Research in Digestive Diseases, Valdecilla Research Institute (IDIVAL), Marqués de Valdecilla University Hospital, 39011 Santander, Spain; malonso@idival.org (M.A.-P.);; 2 Biomedical Research Networking Center in Hepatic and Digestive Diseases (CIBERehd), 28029 Madrid, Spain

**Keywords:** fatty liver, steatohepatitis, personalized medicine, patient phenotyping

## Abstract

Non-alcoholic fatty liver disease (NAFLD) encompasses a spectrum of disorders ranging from simple steatosis to non-alcoholic steatohepatitis (NASH). Hepatic steatosis may result from the dysfunction of multiple pathways and thus multiple molecular triggers involved in the disease have been described. The development of NASH entails the activation of inflammatory and fibrotic processes. Furthermore, NAFLD is also strongly associated with several extra-hepatic comorbidities, i.e., metabolic syndrome, type 2 diabetes mellitus, obesity, hypertension, cardiovascular disease and chronic kidney disease. Due to the heterogeneity of NAFLD presentations and the multifactorial etiology of the disease, clinical trials for NAFLD treatment are testing a wide range of interventions and drugs, with little success. Here, we propose a narrative review of the different phenotypic characteristics of NAFLD patients, whose disease may be triggered by different agents and driven along different pathophysiological pathways. Thus, correct phenotyping of NAFLD patients and personalized treatment is an innovative therapeutic approach that may lead to better therapeutic outcomes.

## 1. Introduction

Non-alcoholic fatty liver disease (NAFLD) includes a wide spectrum of liver injuries ranging from simple steatosis to non-alcoholic steatohepatitis (NASH), which is characterized by a variable grade of inflammation and hepatocellular damage [1,2] and may further progress to more severe hepatic disorders [3]. NAFLD is a growing contributor to end-stage liver disease and liver transplantation [4]. Additionally, NAFLD exhibits a robust correlation with numerous extra-hepatic metabolic conditions, including type 2 diabetes mellitus (T2DM), obesity, hypertension, cardiovascular disease and chronic kidney disease, among others. Consequently, this elevates the mortality rate associated with the condition [5,6]. Such attributes have led to suggestions for a nomenclature change to metabolic-associated fatty liver disease (MAFLD), which carries significant implications for patient management strategies [7]. However, besides metabolic dysfunction, other diseases result in hepatic steatosis, such as alcohol- and drug-induced liver injury, viral infections and chronic inflammatory diseases. Undoubtedly, this liver condition should no longer be considered a “histological disease” and moved away from the two-stage division into NAFLD and NASH as such categorization may not fully reflect the diverse range of disease progression in response to modifications in the underlying metabolic dysfunction or medical treatments [8]. Furthermore, consensus regarding the NAFLD or MAFLD name has not been achieved among experts. Here, we will use NAFLD nomenclature as a standard, except for research on MAFLD as specified by authors.

**Figure 1:**
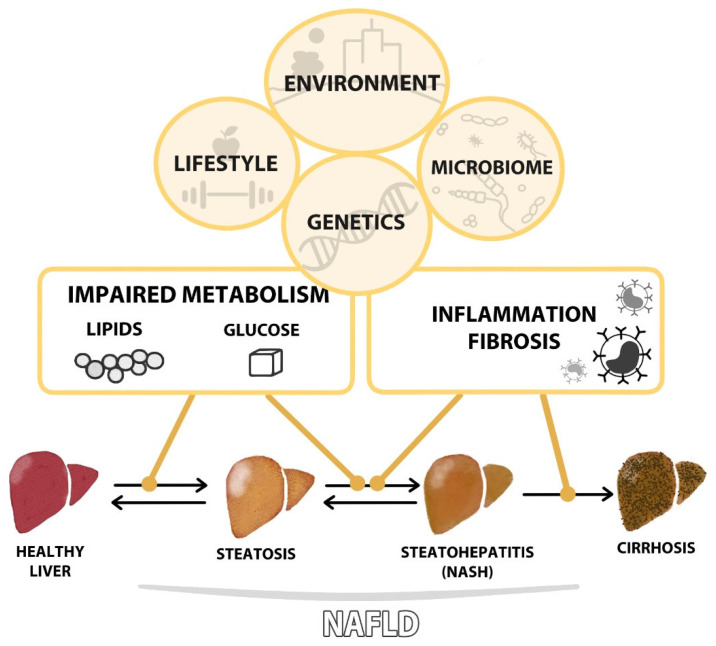
Non-alcoholic fatty liver disease (NAFLD) includes a spectrum of liver manifestations ranging from steatosis to cirrhosis. Progression of the disease is determined by different pathological pathways, mainly metabolism impairment and liver inflammation, which are influenced by patients’ lifestyles, genetics, microbiomes and environmental factors. Interventions on these factors could promote regression at early stages of the disease.

Two main events have been defined in the pathophysiology of NAFLD: lipid accumulation within the hepatocytes, especially free fatty acids (FFAs), and liver-related innate immune responses [9]. However, inflammation may precede steatosis as inflammatory events may lead to lipid accumulation [10]. Therefore, there are many factors influencing NAFLD initiation and progression: environmental exposure, lifestyle, genetic susceptibility, metabolic status and the microbiome [11]. All these factors could induce either steatosis or inflammation, which further triggers endoplasmic reticulum stress, expression of pro-inflammatory cytokines, oxidative stress, hepatic insulin resistance and apoptosis [9,12,13] (Figure 1). The complex interaction of all these mechanisms suggests the existence of different phenotypes within NAFLD that differ in the molecular pathways altered which could result in different natural history, disease course and clinical outcomes.

This review is aimed at discussing the potential stratification of NAFLD patients to provide personalized health care and treatment by summarizing current knowledge about the characteristics of NAFLD patients that could influence pathophysiology and both liver and extrahepatic manifestations of the disease. Understanding the different phenotypes encompassed under NAFLD diagnosis will improve treatment strategies and foster the identification of successful treatments for this pathology by improving clinical trials’ design and control for individual genetic predisposition, signal transduction, or metabolic profiles.

## 2. How to Phenotype NAFLD Patients

Despite more than a decade of extensive research focusing on NAFLD, there is currently no approved therapy for NASH. The complexity and heterogeneity of NAFLD represent important impediments to the discovery of highly effective drug treatments. In addition, clinical trials are not controlled for individual genetic predisposition or signal transduction or metabolic profiles. Trial recruitment is currently based on liver histologic involvement, but many pathological pathways can lead to the same histological phenotype. Therefore, clinical trial reporting for NAFLD is suboptimal, limiting our understanding [14]. The initial step is trying to change this simplistic view of NAFLD, both in clinical trials and in daily clinical practice. A multi-omics data integration approach for NAFLD patients could help us to properly subphenotype and stratify patients, paving the way for precision medicine in NAFLD.

The importance of the classification of NAFLD patients into different subtypes is reflected in several studies based on metabolomics [15,16]. These authors identified a unique serum metabolomic profile of Mat1a (methionine adenosyltransferase 1A) knockout (KO) mice and 0.1MCD (methionine and choline deficient) model and observed, using a large cohort of serum samples from biopsied NAFLD patients, that some of them showed this metabolic signature (M-subtype), identifying those patients that will likely benefit from therapy with S-adenosylmethionine (SAMe) or Aramchol. However, this approach also results in a certain number of unclassified patients (denominated indeterminate). Potential integration of other omics data as well as clinical parameters may improve this novel subtyping approach for NAFLD patients, allowing further interpretation of the complex and heterogeneous disease. Multi-omics observations could identify how genetic, epigenetic, or transcriptional changes lead to metabolic alterations in complex diseases such as NAFLD in a comprehensive manner [17]. Therefore, a comprehensive landscape of the main NAFLD drivers and patient outcome determinants may be obtained by integrating multi-omics and clinical data.

**Figure 2:**
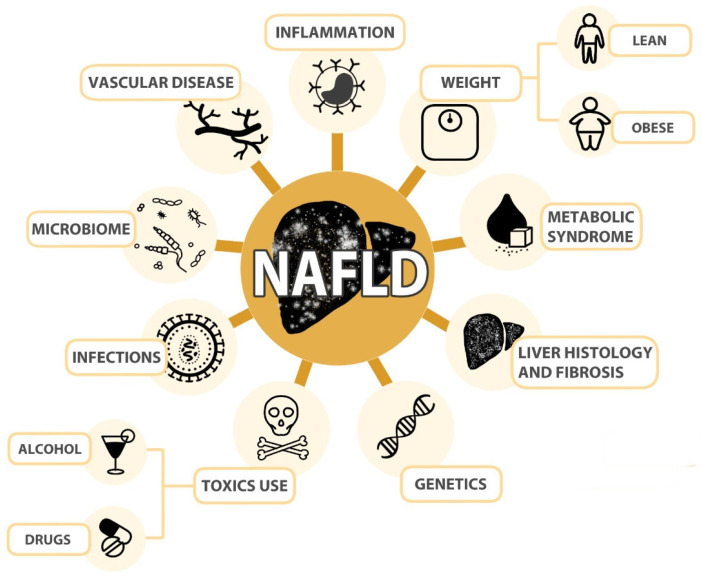
Non-alcoholic fatty liver disease (NAFLD) includes a wide spectrum of patients whose phenotype is determined by the different affection of key processes involved in the pathophysiology of the disease, including body weight and composition, the presence of metabolic syndrome and type 2 diabetes mellitus, liver histological features, genetic predisposition, the use of toxic substances such as small amounts of alcohol and steatogenic drugs, infections and alterations in the gut microbiome, development of vascular disease and systemic inflammation.

Following state-of-the-art research, in this section, we summarize the main factors to take into consideration when attending NAFLD patients, which could lead to better clinical management and treatment (Figure 2).

### 2.1. Histological Features

It is widely accepted that information about disease activity and, in particular, the extent of liver fibrosis is necessary to assess the severity of liver disease and provide prognosis in NAFLD [18]. Histological evaluation of NAFLD liver biopsies is the gold-standard technique for the assessment of disease phenotype and progression [4]. The histological scoring system for staging fibrosis ranges from stage 0 (no fibrosis) to stage 4 (cirrhosis) [18]. However, non-invasive, point-of-care techniques such as imaging modalities (i.e., vibration-controlled transient elastography [VCTE]) and index-based approaches (i.e., FIB-4, NAFLD fibrosis score [NFS]) have been proposed as better options for surveillance programs in at-risk or global populations [19].

It has been shown that the incidence of liver-related complications and all-cause mortality increased with the degree of fibrosis, with fibrosis grades F3 and F4 being associated with an increased risk of liver complications and all-cause mortality [4]. These findings provide support for the use of “progression to cirrhosis” as a generally accepted surrogate outcome for regulatory approval of therapeutic agents. Moreover, the higher rate of hepatic decompensation events (i.e., ascites, variceal bleeding, and encephalopathy) and hepatocellular carcinoma (HCC) among patients with F3 provides a rationale to test the hypothesis that a one-stage regression of fibrosis may translate to fewer hepatic decompensation events [4]. However, fibrosis worsened by one stage (from baseline stage 0 fibrosis) on average during 7.1 years for patients with NASH and by one stage over 14.3 years for patients with steatosis [18]. This long progression time precludes the usefulness of “progression to cirrhosis” as a surrogate outcome as clinical trials are not usually designed for the evaluation of impact over such long periods. In fact, one-stage regression of fibrosis has not been met in most clinical trials testing pharmacological treatments for NAFLD [20]. Furthermore, the incidence of non-hepatic cancers is similar across all fibrosis grades [4], although the leading cause of death in patients with NAFLD is cardiovascular disease, followed by extrahepatic malignancy [18].

On the other hand, the liver phenotype in NAFLD is defined by histological findings such as hepatocellular ballooning, steatosis grade and lobular inflammation [21]. Currently, histologically assessed hepatocyte ballooning is a key feature used in the discrimination of NASH from steatosis as it is considered a form of hepatocyte injury associated with fibrogenesis. This distinction is key to patient selection for trial enrolment, and it also serves as a surrogate endpoint for drug efficacy assessment [22]. Although it is a well-established way to categorize NAFLD patients, liver histology evaluation shows several limitations that might impact clinical trial efficacy and patient management. This includes great inter- and intra-observer variation in pathologists’ assessment of grade of activity in general and ballooning specifically [22]. In this sense, new techniques have been developed to better determine liver fat content, such as magnetic resonance-based methods [23], meanwhile others are still under development [24].

Vilar-Gómez et al. demonstrated the independent association between the presence of steatosis and all-cause mortality, observing that patients with steatosis had cardiovascular and malignant mortality rates comparable to those of patients with cirrhosis [25]. These results strongly suggest that patients with severe steatosis have a higher vascular risk than other patients. Thus, clinicians should pay attention not only to patients with advanced fibrosis but also to those patients with moderate or mild fibrosis who have a high degree of steatosis.

Therefore, although fibrosis assessment and liver phenotyping are essential for the prognosis of NAFLD, they are insufficient for the correct characterization and management of NAFLD patients, and new approaches should be developed to overcome the limitations of histology evaluation.

### 2.2. Metabolic Comorbidities

It has now been established that the primary causes of mortality in patients with NAFLD are cardiovascular disease (CVD) and malignancies, while liver-related mortality occupies the third position. This finding indicates that NAFLD functions as a systemic disorder, which is not unexpected considering its association with insulin resistance (IR) and metabolic syndrome (MetS) [26,27].

NAFLD is closely and bidirectionally associated with MetS and especially with T2DM, whose pathophysiological components are key modifiers of NAFLD development and progression [28]. A recent study published by Ajmera et al. [29] has shown that the prevalence of NAFLD rises to 65% in T2DM patients. Moreover, the global prevalence of NASH rises to 30–40% and significant fibrosis (F2–F4) to 12–20% [30]. Additionally, current clinical evidence highlights that NAFLD could be a precursor to the future development of MetS components and thus linked to an increased cardiovascular risk (CVR) independently of MetS risk factors [31]. Therefore, the association between NAFLD and T2DM brings an additional risk of both hepatic and cardiovascular adverse clinical outcomes; thus, hepatologists should routinely screen for T2DM and perform cardiovascular risk work-ups periodically [32].

We should note that the link between T2DM and NAFLD is complex [33]. These pathologies share many risk factors, such as impairment of glucose and lipid metabolism, and NAFLD is also predictive of T2DM (see review by Muzica et al. [34]). In contrast to other T2DM complications, screening and assessment of NAFLD are not usually routinely done [32]. However, as T2DM may promote progression to NASH and liver fibrosis in up to 20% of the patients [35,36,37], which can eventually turn into liver cirrhosis and/or HCC, screening for NAFLD should be done in these patients. The latest American Association of Clinical Endocrinology recommendation is that patients with T2DM or prediabetes and elevated liver enzymes or fatty liver disease on ultrasound should be evaluated for the presence of NAFLD [37]. These individuals should be screened for liver fibrosis using non-invasive methods, such as FIB-4 and NFS, followed by VCTE or analysis of patented serum biomarkers [34].

### 2.3. Weight

#### 2.3.1. Obese Patients

NAFLD is the most frequent cause of chronic liver disease, with a global prevalence around 25% [38,39]. However, this percentage is increased in obese patients for whom the prevalence rises to 58% [39]. In obese children and adolescents, the prevalence is also high (44%), being greater in developed countries. Moreover, the prevalence increases as does the Body Mass Index (BMI): being 20.23% in overweight and 38.47% in obese patients [40].

The underlying pathophysiology is based on the presence of IR and adipocyte dysfunction, which lead to lipolysis, with an increase in circulating FFAs and leptin and a decrease in adiponectin, which favors intrahepatic fat accumulation. This fact is worsened with de novo lipogenesis because of fat and carbohydrates in the diet. Moreover, immune cells can infiltrate the liver, further producing a chronic low-grade intrahepatic inflammation. Lipotoxicity and glucotoxicity along with mitochondrial damage and oxidative stress lead to NASH progression and ultimately to the development of fibrosis [41].

Thus, obese patients have greater liver impairment with higher aspartate aminotransferase (AST) and alanine aminotransferase (ALT) levels and more liver fibrosis [42]. The presence of obesity in NAFLD is associated with the occurrence of hypertriglyceridemia (OR 1.51), MetS (OR 2.66), hypertension and T2DM (OR 1.35) [42]. That is, it is associated with a higher prevalence of other CVR factors [43].

As discussed later, diet and exercise are the main treatment of patients with NAFLD and obesity [44]. Weight loss of ≥10% induces high rates of improvement (>80%) not only of the comorbidities but also of all the histological lesions of NAFLD [25].

#### 2.3.2. Lean Patients

Although NAFLD has a strong association with obesity, there is a proportion of cases with a normal BMI, which is usually called “non-obese NAFLD” or “lean NAFLD”. These two terms are not exactly synonyms and vary between studies: the first includes both overweight and normal weight patients and the second, normally, only those with normal BMI [45].

In a recent meta-analysis, the global prevalence of non-obese NAFLD was 40.8% within the NAFLD population and 12.1% within the general population. However, the prevalence varied by BMI, being more frequent in overweight than in normal weight people. Thus, the prevalence of lean NAFLD turned out to be 19.2% in the NAFLD population and 5.1% within the general population [46].

It is important to mention that a normal BMI is not a synonym for a metabolically healthy condition. People with lean MAFLD have lower waist circumference, diastolic blood pressure and serum triglycerides (TG) compared to overweight/obese MAFLD patients. Moreover, they have a different body composition, with significantly lower fatty tissue index, lean tissue index and total body water. However, compared to lean healthy controls, lean MAFLD had a worse metabolic profile, characterized by a higher percentage of hypertension, BMI, TG, low-density lipoprotein (LDL), glucose, HbA1C and lower HDL [47].

It has been stated that lean NAFLD patients have a less severe disease than obese NAFLD ones. In a prospective study, non-obese NAFLD patients seem to have lower NAFLD activity scores (NAS) than obese NAFLD ones, mainly attributable to lesser steatosis and a smaller proportion of ballooning. Moreover, non-obese NAFLD patients had less fibrosis. When analyzing the different parameters of MetS, it was observed that only TG levels were an independent predictor of disease severity [48]. In a retrospective Italian study, similar results were found, with significantly lower proportions of NASH (17% vs. 40%) patients and significant fibrosis (i.e., >F2) (17 vs. 42%) among lean NAFLD patients in comparison to overweight or obese NAFLD patients [49]. However, the presence of MetS seems to be associated with the progression to NASH and significant fibrosis in patients with NAFLD regardless of BMI [50]. In a large retrospective cross-sectional study in Asia, it was also found that MetS in non-obese NAFLD was associated with NASH (OR 1.59) and advanced fibrosis (OR 1.88) [51].

On the other hand, it was classically considered that lean NAFLD patients had a more benign course of illness, with a lower incidence of new onset CVD. However, it has been observed that all-cause cardiovascular and hepatic mortality are not negligible in these patients (incidence per 1000 person–years of 12.1%, 4% and 4.1%, respectively). Although it should be noted that non-obese NAFLD (i.e, patients with normal weight but also overweight) were included in this meta-analysis [46]. In a population-based cross-sectional study carried out in Korea, lean NAFLD patients seemed to have a significantly higher atherosclerotic cardiovascular disease (ASCVD) score and prevalence of a high ASCVD risk compared to obese NAFLD patients. However, this study had some limitations: first, being a cross-sectional study, longitudinal follow-up is not possible; second, the severity of the disease was measured by indirect methods, without biopsy; and third, cardiovascular events were not evaluated [52]. In a retrospective study, one-third of the lean NAFLD patients had carotid atherosclerosis [49].

The pathophysiology of lean NAFLD is not fully understood yet, with multiple factors that can have influence having been described [53]. It may be set by the genetic background and early alterations in bile acid (BA) and gut microbiota profile. Thus, lean NAFLD had a higher chance of carrying at least one PNPLA3 risk allele compared to lean healthy controls [42,54]. Lean NAFLD patients had higher total, primary and secondary BA levels than overweight–obese NAFLD ones, although it was only significant for secondary BAs. Moreover, the composition was different as lean NAFLD patients had lower deoxycholic acid, glycochenodeoxycholic acid and chenodeoxycholic acid but more glycocholic acid [55]. On the other hand, a lower level of various lysophosphatidylcholines, which is linked to obesity and hypertriglyceridemia, has been described in lean NAFLD patients [54].

All in all, the latest guidelines recommend that even with a normal weight, lean NAFLD patients should undergo lifestyle intervention, including exercise, diet modification, and avoidance of fructose- and sugar-sweetened drinks, to target a modest weight loss of 3–5% [56].

### 2.4. Cardiovascular Diseases

NAFLD patients are at risk of cardiovascular and cardiac diseases. They have more subclinical atherosclerosis, arrhythmias, cardiovascular events, conduction defects, aortic-valve sclerosis and heart failure, which increase disease-related morbimortality [57].

It seems that the metabolic dysfunction that defines MAFLD is associated with higher risks of all-cause mortality and cardiovascular mortality in MAFLD patients compared to patients with NAFLD [58].

In a recent study, three subtypes of NAFLD patients were identified. Subtype A, which phenocopied the metabolome of mice with impaired VLDL-TG secretion, had the lowest CVR measured by Framingham risk score. Moreover, it had lower serum TG, cholesterol, VLDL, small dense LDL and remnant lipoprotein cholesterol compared to type B (intermediate) and C (normal VLDL-TG) [59]. Thus, CVR assessment is a priority. ASCVD can be an easy tool as an ASCVD score ≥ 7.5% was associated with a higher risk of overall and cardiac-specific mortality [60].

In a large cohort study with more than 10,000 NAFLD patients, it was described that NAFLD subjects tended to meet a lower number of ideal health metrics (BMI, smoking, physical activity, diet, blood pressure, cholesterol and glycemia). If these modifiable risk factors were addressed, 66% of all-cause deaths and 83% of cardiovascular deaths were preventable [61].

### 2.5. Inflammatory Dysfunction

Growing evidence has pointed towards a disproportionately high prevalence of NAFLD and advanced fibrosis in patients with immune-mediated inflammatory diseases (IMID) such as psoriasis [62], inflammatory bowel disease (IBD) [63] and hidradenitis suppurativa [64]. Although this may be explained by an interplay between the distinctive chronic inflammation of IMIDs and the co-existence of metabolic risk factors, the IMID diagnosis acts as an independent risk factor for NAFLD. For instance, in IBD patients, advanced fibrosis was particularly prevalent, regardless of the influence of metabolic risk factors [63]. Therefore, while advanced fibrosis was found to be three times more common in NAFLD–IBD individuals than in the general population with NAFLD, the disparities were even greater when obesity was not present, with a four-fold higher prevalence. Furthermore, in the absence of T2DM, the prevalence of advanced fibrosis was nearly five times higher in the IBD population, and in individuals without both obesity and T2DM, the difference was almost seven times as great [63].

Thus, NAFLD has a disproportionately high tendency to develop in IMID populations, which may be explained by the distinctive chronic inflammatory burden of these conditions [63]. Interestingly, NASH and most IMIDs share some molecular characteristics such the activation of the tumor pathways depending on tumor necrosis factor (TNF)-α or the imbalance in T-cell subtypes such as Th17/Treg. This common pathogenesis may explain, at least in a subset of patients, the development of NASH in the absence of classic metabolic risk factors [64]. Spanish guidelines are moving towards the inclusion of these patients in NAFLD screening strategies [65]. However, international guidelines have not yet recognized the need for NAFLD evaluation in IMID patients [66] and studies regarding the link between NAFLD and IMID pathogenesis are still pending.

### 2.6. Genetic Factors

Genetic and epidemiological studies indicate strong heritability of hepatic fat content and the key role of genes in the development and progression of liver diseases [67]. In particular, genome-wide association studies (GWAS) have shown a significant relation between several single nucleotide polymorphisms (SNPs) and an increased risk of chronic liver disease [68]. In fact, there are several polymorphisms in different genes associated with NAFLD development and progression. The five more strongly related with disease severity are: patatin-like phospholipase domain (*PNPLA3*), transmembrane 6 superfamily member 2 (*TM6SF2*), glucokinase regulator (*GCKR*), membrane bound O-acyltransferase domain-containing 7 (*MBOAT7*) and hydroxysteroid 17β-dehydrogenase (*HSD17B13*) [69] (Table 1).

One of the most described and robustly validated associations is a missense variant in *PNPLA3*. The substitution of cytosine by guanine in codon 148 results in an amino acid change from isoleucine to methionine in PNPLA3 (rs738409; p.Ile148Met), which is strongly associated with hepatic fat content and inflammation as described by Romeo, S. et al. [70]. PNPLA3 protein is implicated in lipid regulation in hepatocytes and stellate cells. In hepatocytes, PNPLA3 acts as a triacylglycerol lipase and acylglycerol O-acyltransferase which involves catalyzing the transfer of polyunsaturated fatty acids (PUFA) from di- and tri-acylglycerols to phosphocholines [88].

PNPLA3 is degraded through ubiquitination of lysine and subsequent proteosome degradation. The lack of function in *PNPLA3* rs738409 and its loss of accessibility to be ubiquitinated leads to a retention of TG and PUFA in the liver [89]. In NAFLD patients, the phenotypic manifestations of this polymorphism are higher TG levels, elevated ALT and AST ratio, severity of steatohepatitis and increased fibrosis [68].

A missense variant in *TM6SF2*, encoding transmembrane 6 superfamily member 2, is associated with NAFLD. *TM6SF2* is mainly expressed in the liver and small intestine, although its exact function is not well known; TM6SF2 regulates fat metabolism, specifically cholesterol synthesis and lipoprotein secretion [90]. The rs58542926 polymorphism encodes a substitution of glutamic acid to lysine at position 167 (p.Glu167Lys). This amino acid change results in a loss-of-function, inducing higher liver TG levels and lower circulating lipoproteins [69].

In vitro studies revealed that *TM6SF2* siRNA inhibition was associated with the reduced secretion of very-low density lipoproteins (VLDLs) and increased cellular TG concentrations and lipid droplet levels, whereas *TM6SF2* overexpression reduced liver cell steatosis [76]. Apparently, *TM6SF2* rs58542926 polymorphism elevates the risk of liver disease but reduces cardiovascular event risk [91].

*GCKR* controls de novo lipogenesis by regulating the influx of glucose into hepatocytes [78]. Loss-of-function in *GCKR* (rs1260326; p.Pro446Leu) regulates glucokinase in response to fructose-6-phosphate, activating hepatic glucose uptake. This leads to decreased circulating fasting glucose and insulin levels but increases the production of malonyl-CoA. In fact, a higher concentration of malonyl-CoA favors hepatic fat accumulation by serving as a substrate for lipogenesis and by blocking fatty-acid β-oxidation in the mitochondria [68,92]. Thus, rs1260326 has been related to NAFLD [78].

Under the hypothesis that alcoholic liver disease (ALD) and NAFLD share common genetic determinants, Mancina et al. [93] identified a significant *locus* for both pathologies using GWAS. *MBOAT7* is highly expressed in inflammatory and immune cells. It encodes lysophosphatidylinositol acyltransferase 1 (LPIAT1), which is involved in remodeling arachidonic acid to phosphatidynositol in the Lands cycle [72]. A study in a European descent cohort demonstrated the association between *MBOAT7* rs641738 and the development and severity of NAFLD [93].

The latest addition to the list of genes that are involved in NAFLD, based on GWAS studies, was HSD17B13, a liver-specific enzyme that regulates lipid homeostasis. Its aberrant expression and high enzyme activity have been confirmed to promote the development of NAFLD [73]. In contrast, the polymorphism *HSD17B13* rs72613567 results in a loss-of-function truncated protein, thus attenuating the progression of NAFLD. Furthermore, *HSD17B13* rs72613567 has been associated with reduced serum AST and ALT levels, lower inflammation and NAS including ballooning and fibrosis [94]. These results allow researchers to conclude that the truncated protein has a protective role against liver diseases [95].

### 2.7. Microbiome

The research carried out in recent years points to the fundamental role of the gut microbiome (GM) in the development of NAFLD as well as in multiple physiological processes such as energy metabolism and immune functions [96].

The human GM is dominated by four bacterial phyla: Bacteroidetes, Firmicutes, Proteobacteria and Actinobacteria [97]. Recent studies show that lean and obese individuals differ in gut bacterial composition [98]. In fact, obesity has been associated with phylum-level changes in the GM, reduced bacterial diversity and altered representation of bacterial genes and metabolic pathways [99]. In NAFLD, it has been demonstrated that alterations in the GM go through an increase in Gram negatives and a decrease in Gram positives, which translates to an increase in Proteobacteria and a decrease in Bacteroidetes-Firmicutes ratio at the phylum level [100].

Dysbiosis has been described as an imbalance in the microbiota composition and function, resulting in a negative effect on the physiology of the host. It could be caused by several environmental factors, such as diet, physical activity, medication and geographical localization [101,102].

The GM interaction with the liver via the “gut–liver axis”, established by the portal vein, enables the transport of GM-derived products directly to the liver and bile and antibody secretion in the opposite way [103]. Alterations in the gut–liver axis caused by GM imbalance and mucosa permeability changes may allow metabolic bacterial products and components to cross the intestinal barrier and reach the liver, causing inflammatory and oxidative responses and exacerbating NAFLD pathology [104]. Some bacterial metabolites could interfere with glucose and lipid metabolism, triggering liver disease; whereas microbial components, pathogen-associated molecular patterns such as lipopolysaccharide and peptidoglycan, can activate pattern recognition receptors (PRRs) in Kupffer cells and hepatic stellate cells, inducing inflammatory responses and contributing to liver injury and fibrosis [103,105,106]. All these conditions are involved in NAFLD progression [102]. Murine models have given further support to the role of microbiota in liver fibrosis as high-fat diet microbiota transplantation to control mice resulted in an increase in liver injury [107].

Focusing on the role of metabolites in alcohol fermentative pathways, acetaldehyde and acetate have been involved in the degradation of intestinal tight junctions [108,109]. In fact, the genes that encode for these enzymes in the gut microbiome are overexpressed in NAFLD, which suggests that alcohol metabolism could be a trigger in this pathology [110].

The GM is also involved in the fermentation of other compounds such as complex carbohydrates to produce short-chain fatty acids including acetate, propionate and butyrate [111]. It has been described that butyrate contributes to the maintenance of the intestinal barrier and its reduction is related to the weakening of tight junctions and an increase in permeability [112,113].

Intestinal metabolic dysregulation has been associated with the metabolism of aromatic amino acids (AAA). In healthy conditions, the AAA tryptophan can be metabolized by the GM, producing indole. Further studies have demonstrated the beneficial effect of indole and its derivatives in upregulating endothelial tight junctions, downregulating pro-inflammatory cytokines production and modulating the secretion of glucagon-like peptide-1 (GLP-1) [114,115].

Thus, accumulated evidence supports the significant role of microbiome dysbiosis in NAFLD onset and progression and it constitutes an important factor to consider for patient management. Furthermore, therapies targeting dysbiosis are under investigation and NAFLD patients showing this condition are expected to be most benefited by its treatment [116].

### 2.8. Toxics Consumption

#### 2.8.1. Alcohol

NAFLD diagnosis is based on the exclusion of harmful alcohol intake, which has been set as daily ingestion below 20 g (women) and 30 g (men) of pure ethanol by European and American guidelines [117,118]. However, the World Health Organization reported that the average pure ethanol use exceeds those limits (it rises to 32.8 g ethanol/day among women, and more than 40 g ethanol/day among men) [119] and is associated with significant health risks. In fact, moderate (20–40 g ethanol/ day) or heavy (>40 g ethanol/day) alcohol use causes additional liver damage and hepatic steatosis in more than 25% of patients with presumed NAFLD [120,121]. In contrast, there is conflicting evidence of a slightly protective effect of low and moderate alcohol consumption in NAFLD (see review by Petroni et al., 2019 [122]).

The main challenge limiting an accurate diagnosis relies on the fact that NAFLD and ALD have not been reliably distinguished by well-established diagnostic means. Using non-invasive biomarkers, such as ethyl glucuronide (EtG, a metabolite of alcohol), in hair and urine can accurately detect potentially harmful alcohol consumption in patients with NAFLD [120,123]. Hence, according to hair EtG levels, presumed NAFLD patients can be reclassified with regard to their risk of alcohol-related liver damage due to repeated moderate–excessive alcohol consumption [120]. Lifestyle intervention in NAFLD patients with low alcohol consumption should include the recommendation of total abstinence.

#### 2.8.2. Drugs

Drug-induced steatosis (DIS) is usually associated with the prolonged intake of a medication at a specific dose, and it is relatively rare as just 2% of NAFLD cases are estimated to be drug-induced [124]. This phenomenon involves an acute energy crisis through inhibited fatty acids β-oxidation and other impaired mitochondrial and peroxisomal functions, initially resulting in microvesicular steatosis that can usually be reversed (reviewed by Dash et al. [125]). Table 2 summarizes the most commonly used drugs known to cause steatosis.

Imaging methods can estimate hepatic fat content, but these techniques are unreliable when distinguishing steatosis from steatohepatitis [151,152]. Furthermore, serum transaminases’ usefulness as noninvasive indicators of DIS is limited since these proteins are within the reference range in most individuals with hepatic steatosis [153,154,155]. Hence, finding sensitive and specific serum biomarkers is key to assessing the contribution of drugs to NAFLD. Cytokeratin 18 (CK18), fibroblast growth factor 21 (FGF21), insulin-like growth factor binding protein 1 (IGFBP1), several microRNAs and forkhead box protein A1 (FOXA1) have been identified as potential biomarkers for DIS detection and prognosis, as reviewed by Pavlik et al. [156].

### 2.9. Concomitant Infections

Since the improvement of antiretroviral regimens, NAFLD has emerged as a growing concern in the long-term management of patients with HIV mono-infection. HIV infection itself induces various metabolic alterations that can lead to steatosis by disrupting fatty acid β-oxidation in the liver and adipose tissue [157,158]. Nevertheless, and as mentioned above, it should also be noted that antiretroviral therapy may be partially responsible for this steatogenic effect. Moreover, HIV invasion of hepatic stellate cells triggers fibrosis [159,160], favoring liver damage progression to NASH.

Several studies have attempted to determine the prevalence of NAFLD or NASH in HIV-infected patients, and those have been reviewed in detail by authors such as Verna [161], Squillace et al. [162], and Morrison et al. [163]. However, the definition of NAFLD, study populations, group matching criteria, and methods for fatty liver assessment are heterogeneous, making the outcomes difficult to interpret or even contradictory among different studies. Previous results suggest that NAFLD prevalence in HIV-infected individuals is higher (30–50%) and progresses at an increased rate compared to the general population, with antiretroviral exposure being an additional risk factor [164,165]. Lipodystrophy syndrome, which includes abnormal fat distribution and increased visceral adiposity, is usually present in HIV-positive patients [166,167] and it directly contributes to NAFLD development [164,165].

HIV-induced steatosis and/or fibrosis can be detected through imaging techniques [168,169] and ultrasound/transient elastography [170,171,172]. Regarding blood tests, HIV-positive patients at risk of NASH may show increased levels of serum transaminases [173,174,175] and, more precisely, higher scores for non-invasive markers of fibrosis (FIB-4, APRI) [169,176,177,178,179,180] compared to HIV-negative patients. However, these tests might not be accurate enough, so there is an urgent need to develop and validate new non-invasive biomarkers and imaging assessments for liver disease in HIV-positive patients. Recently, several proteins have aroused interest as biomarkers for detecting steatosis (FGF21 [181], IL-18 [182]) and fibrosis (CK18 [183]), as well as other proteins involved in tissue repair and immune response pathways [184] in these individuals. In addition, some polymorphisms may predict NAFLD development in HIV-infected patients [185], and there is an increasing focus on circulating miRNAs as a non-invasive reflection of liver disease progression in people living with HIV (thoroughly reviewed by Martinez et al. [186]).

## 3. NAFLD Treatment

### 3.1. Treatments to Rule Them All

Lifestyle intervention, with modifications in diet and physical activity, has become the first line treatment for patients with NAFLD. A greater extent of weight loss, induced by lifestyle changes, is associated with the level of improvement in histologic features of NASH. The highest rates of NAS reduction, NASH resolution and fibrosis regression occurred in patients with weight losses of 10% or more [25]. Furthermore, age, sex, T2DM and genes impact on the effect of diet in weight loss and NASH resolution [187]. These factors are integrated in the so called ‘nutritional geometry’, which considers the relevance of nutrition, science and the environment to understand how food components interact to regulate the properties of diets [188]. Stratifying patients according to the geometry of nutrition could improve the rate of response [187].

Regarding weight loss, therapies such as bariatric surgery and metabolic endoscopic techniques can be useful alternatives as only 10% of participants achieved enough body weight reduction through lifestyle interventions [189]. Bariatric surgery has also been shown to improve obesity, its metabolic consequences and NASH [190,191]. However, given the surgical risk, it cannot be considered a first-line therapy, especially in those patients with decompensated cirrhosis or portal hypertension. In this context, endoscopic bariatric techniques have emerged as a potential treatment option as they can reproduce those benefits in a minimally invasive manner [192]. However, this kind of intervention might be eligible only in obese patients.

On the other hand, a recent expert meeting has gathered strong evidence that regular physical activity plays an important role in preventing NAFLD and improving intermediate clinical outcomes [193]. Various studies have demonstrated an improvement in NAFLD with personalized physical exercise programs [194], even in the absence of significant weight loss [195,196], and a reduction of the hepatic venous pressure gradient in cirrhotic patients [197].

The prescription of an appropriate diet and the indication of physical exercise in proportion to the disease and the characteristics of the patients is the main curative option for all NAFLD patients, including lean NAFLD [55,198]. A randomized controlled trial from Asia demonstrates using MR spectroscopy that almost half of non-obese individuals achieved NAFLD remission with 3–5% weight loss [198].

Thus, an innovative therapeutic strategy in this setting would be the constitution of multidisciplinary units integrating clinicians (hepatologists, endocrinologists, cardiologists, internists), physiotherapists, nutritionists, nurses, social educators and, of course, patients, where a health-promoting diet, avoidance of tobacco, alcohol and other toxins, and sustainable, inclusive and adapted physical activity is prescribed considering the needs of each patient. Furthermore, such multidisciplinary units allow the integration of adequate assessments for the risk of both significant liver and vascular disease, macro and/or microvascular complications of T2DM, the risk of hepatic and extrahepatic neoplasms and other potential comorbidities.

### 3.2. Targeted Therapy

Thanks to the continuous research on NAFLD pathogenesis, several druggable targets have been identified and thus targeted therapies for NAFLD treatment have entered clinical trials, which have been recently and extensively reviewed by Santos-Laso et al. [199]. However, limited impact has been achieved for now, probably due to the extensive placebo effect in NAFLD [200], the complexity of the pathological pathways involved [189], the short follow-up time for the expected outcomes to be evaluated and the limited characterization of NAFLD patients before inclusion in clinical trials [201].

Table 3 summarizes the main targeted therapies for NAFLD which are currently under evaluation in Phase III clinical trials.

#### 3.2.1. Targeting Lipid Metabolism

Peroxisome proliferator-activated receptors (PPARs) are ligand-activated transcription factors which include PPARα, PPARγ and PPARβ/δ. The pan-PPAR agonist lanifibranor acts through the activation of all PPAR isoforms, reducing TG levels and increasing insulin sensitization, glucose metabolism and fatty acid metabolism. Lanifibranor has successfully completed a 24-week phase IIb trial, meeting its primary endpoint of a reduction of two points or more in the SAF (steatosis, activity and fibrosis) score, with no increase in fibrosis, and the secondary endpoint of reducing fibrosis by at least one stage without worsening NASH. Lanifibranor is well tolerated, although side effects include mild weight gain. Thus, a phase III study is underway [20].

Statins have been demonstrated to reduce steatosis in those with NASH and it prevents liver events in patients with metabolic syndrome with advanced NASH. Furthermore, a possible protective role of statin treatment against NAFLD progression to HCC was also demonstrated in observational studies. Interestingly, it has been shown that statins reduce CVR more in NAFLD vs. non-NAFLD high-risk individuals [32]. Thus, evaluation in a phase III clinical trial of rosuvastatin treatment for NAFLD is about to start recruitment (Table 3).

Oltipraz is a synthetic dithiolethione that functions as an anti-steatogenic agent against NAFLD by inhibiting LXR-α activity, which decreases the expression of SREBP-1c within the liver, reducing the synthesis of fatty acids but enhancing lipid oxidation [202]. Although two phase III clinical trials have been completed, no data are available yet (Table 3). However, the results from the phase II trial showed that oltipraz decreased liver fat content and BMI while absolute changes in insulin resistance, liver enzymes, lipids and cytokines were not significant [202].

Resmetirom is a liver-directed, orally active, selective thyroid hormone receptor-β agonist designed to improve NASH by increasing hepatic fat metabolism and reducing lipotoxicity. In phase II clinical trials, Resmetirom treatment resulted in significant reductions in hepatic fat after 12 weeks and 36 weeks of treatment in patients with NASH, while the adverse events were transient mild diarrhea and nausea [203]. Two phase III trials are currently recruiting participants (Table 3).

It could be hypothesized that obese patients, those with genetic predisposition to lipid accumulation or increased circulating levels of TG due to metabolic syndrome, might benefit from therapies targeting lipid metabolism.

#### 3.2.2. Targeting Glucose Metabolism

Pioglitazone is a PPARγ agonist used in the treatment of T2DM due to its properties as an insulin sensitizer [204]. Moreover, results have been published regarding pioglitazone evaluation in non-diabetic NAFLD patients in comparison to vitamin E supplementation [205]. There was no benefit of pioglitazone compared to placebo for the primary outcome; however, significant benefits of pioglitazone were observed for some of the secondary outcomes such as steatohepatitis resolution, decrease in mean AST and ALT levels and improvement in insulin resistance [205].

GLP-1 receptor agonists (GLP-1RA) are widely used in the treatment of T2DM. Studies have found that GLP-1R has multiple biological effects, such as neuroinflammation reduction, nerve growth promotion, heart function improvement, appetite suppression, gastric emptying delay, blood lipid metabolism regulation and fat deposition reduction. Thus, effects of GLP-1RA include neuroprotection, cardiovascular protection and metabolic regulation [206]. Semaglutide has completed a phase II trial showing resolution of NASH with no worsening of fibrosis. Although it was unable to achieve its secondary outcome of improvement of fibrosis with no worsening of NASH, the drug induces a significant weight loss and most common adverse events were gastrointestinal [20]. Encouraging pilot results of the evaluation of exenatide, another GLP-1RA, for the treatment of NAFLD have been released [207]. Moreover, cotadutide, a dual GLP-1RA and glucagon receptor agonist, has been evaluated in a phase IIb clinical trial showing improved glycemic control and weight loss, along with improvements in hepatic parameters such as reduction in ALT, AST and gamma-glutamyltransferase (GGT) levels, as well as improvements in NFS and FIB-4 index [208].

Dipeptidyl peptidase 4 (DPP-4) inhibitors work by blocking the enzymatic inactivation of endogenous incretin hormones, resulting in glucose-dependent insulin release and a decrease in glucagon secretion [32]. Early results evaluating DPP-4 inhibitor vildagliptin in NAFLD patients with T2DM have shown significant improvement in blood sugar regulation, BMI, ALT, liver fibrosis and steatosis indices [209].

SGLT-2 inhibitors promote urinary excretion of glucose by inhibiting its renal proximal tubular reabsorption [32]. Dapagliflozin and empagliflozin are SGLT-2 inhibitors undergoing phase III clinical trials for the treatment of NASH. To date, it has been demonstrated that dapagliflozin can markedly reduce hepatic enzymes and metabolic indicators and improve body composition [210].

These drugs are prescribed in the treatment of T2DM. Thus, clinical guidelines have already included the preferential use of drugs with effects on the liver in the management of T2DM patients with NAFLD [211].

**Table 3:** Targeted drugs for the treatment of adult NAFLD being evaluated in phase III clinical trials [212].

Mechanism	Drug	Identifier	Intervention	Title	Status
Lipid metabolism	Lanifibranor	NCT04849728	Drug: Lanifibranor|Drug: Placebo	A Phase 3 Study Evaluating Efficacy and Safety of Lanifibranor Followed by an Active Treatment Extension in Adult Patients With (NASH) and Fibrosis Stages F2 and F3 (NATiV3)	Recruiting
	Rosuvastatin	NCT05731596	Drug: Rosuvastatin 20 mg Oral Tablet|Drug: Coenzyme Q10 100 mg Oral Capsule	Comparative Clinical Study to Evaluate the Efficacy and Safety of Rosuvastatin vs. CoQ10 on Non-alcoholic Steatohepatitis	Not yet recruiting
	Oltipraz	NCT04142749	Drug: Oltipraz|Drug: Placebos	Oltipraz for Liver Fat Reduction in Patients with Non-alcoholic Fatty Liver Disease Except for Liver Cirrhosis	Completed
		NCT02068339	Drug: Oltipraz 1 (90 mg)|Drug: Placebo|Drug: Oltipraz 2 (120 mg)	Efficacy and Safety of Oltipraz for Liver Fat Reduction in Patients with Non-alcoholic Fatty Liver Disease Except for Liver Cirrhosis	Completed
	Resmetirom	NCT03900429	Drug: Resmetirom|Drug: Placebo	A Phase 3 Study to Evaluate the Efficacy and Safety of MGL-3196 (Resmetirom) in Patients with NASH and Fibrosis	Recruiting
		NCT04197479	Drug: Placebo|Drug: Resmetirom	A Phase 3 Study to Evaluate the Safety and Biomarkers of Resmetirom (MGL-3196) in Non-Alcoholic Fatty Liver Disease (NAFLD) Patients	Active, not recruiting
Glucose metabolism	Pioglitazone	NCT05521633	Drug: Metformin and Pioglitazone	Comparison of the Effects of Metformin and Pioglitazone on Liver Enzymes and Ultrasound Changes in Non-Diabetic Non-alcoholic Fatty Liver	Completed
		NCT05605158	Drug: Pioglitazone 30 mg|Drug: Empagliflozin 10 mg	Comparative Clinical Study Between Empagliflozin Versus Pioglitazone in Non-diabetic Patients with Non-alcoholic Steatohepatitis	Not yet recruiting
		NCT00063622	Drug: Pioglitazone|Dietary Supplement: Vitamin E|Drug: Matching placebo	Pioglitazone vs. Vitamin E vs. Placebo for Treatment of Non-Diabetic Patients with Non-alcoholic Steatohepatitis (PIVENS)	Completed
	Semaglutide	NCT05067621	Drug: Semaglutide Pen Injector|Drug: Placebo	Semaglutide Effects in Obese Youth with Prediabetes/New Onset Type 2 Diabetes and Non-alcoholic Fatty Liver Disease	Not yet recruiting
		NCT03919929	Drug: Semaglutide 3 mg and 7 mg [Rybelsus]|Other: Weight loss diet	Treating PCOS With Semaglutide vs. Active Lifestyle Intervention	Recruiting
		NCT04822181	Drug: Semaglutide|Drug: Placebo	Research Study on Whether Semaglutide Works in People with Non-alcoholic Steatohepatitis (NASH)	Recruiting
	Exenatide	NCT00650546	Drug: Exenatide	Role of Exenatide in NASH-a Pilot Study	Completed
	Cotadutide	NCT05364931	Drug: Cotadutide|Drug: Placebo	A Study to Evaluate the Safety and Efficacy of Cotadutide Given by Subcutaneous Injection in Adult Participants with Non-cirrhotic Non-alcoholic Steatohepatitis With Fibrosis.	Active, not recruiting
	Vildagliptin	NCT03925701	Drug: Vildagliptin|Drug: vildagliptin\metformin	Clinical Study Evaluating Vildagliptin Versus Vildagliptin/Metformin on NAFLD With DM	Recruiting
	Dapagliflozin	NCT05308160	Drug: Dapagliflozin 10 mg Tab|Drug: Placebo	A Single Center, Randomized, Open Label, Parallel Group, Phase 3 Study to Evaluate the Efficacy of Dapagliflozin in Subjects with Non-alcoholic Fatty Liver Disease	Recruiting
		NCT03723252	Drug: Dapagliflozin|Drug: Placebo	Dapagliflozin Efficacy and Action in NASH	Recruiting
	Empagliflozin	NCT05605158	Drug: Pioglitazone 30 mg|Drug: Empagliflozin 10 mg	Comparative Clinical Study Between Empagliflozin Versus Pioglitazone in Non-diabetic Patients with Non-alcoholic Steatohepatitis	Not yet recruiting
Bile acid metabolism	Obeticholic acid	NCT02548351	Drug: Obeticholic Acid|Drug: Placebo	Randomized Global Phase 3 Study to Evaluate the Impact on NASH With Fibrosis of Obeticholic Acid Treatment	Active, not recruiting
		NCT03439254	Drug: Obeticholic acid (10 mg)|Drug: Obeticholic acid (10 mg to 25 mg)|Drug: Placebo	Study Evaluating the Efficacy and Safety of Obeticholic Acid in Subjects with Compensated Cirrhosis Due to Non-alcoholic Steatohepatitis	Completed
	Aramchol	NCT04104321	Drug: Aramchol|Drug: Placebo	A Clinical Study to Evaluate the Efficacy and Safety of Aramchol in Subjects with NASH (ARMOR) (ARMOR)	Suspended
Oxidative stress, inflammation and fibrosis	N-acetylcysteine	NCT05589584	Drug: N acetyl cysteine with weight reduction	N-acetyl Cysteine and Patients with Non-alcoholic Fatty Liver Disease	Recruiting
	Pentoxifylline	NCT05284448	Drug: pentoxifylline (Trental SRÂ^®^)	Pentoxifylline in Treatment of Patients with Non-alcoholic Steatohepatitis	Active, not recruiting
		NCT00267670	Drug: Pentoxifylline|Drug: Placebo	Pentoxifylline/Non-alcoholic Steatohepatitis (NASH) Study: The Effect of Pentoxifylline on NASH	Completed
	Secukinumab	NCT04237116	Biological: Investigational Arm—secukinumab|Biological: Control Arm—placebo	A Study of Secukinumab Treatment in Patients with Plaque Psoriasis and Coexisting Non-alcoholic Fatty Liver Disease (NAFLD)	Completed
	Lubiprostone	NCT05768334	Drug: Lubiprostone 24 Mcg Oral Cap	Efficacy and Tolerability of Lubiprostone in Patients with Non-alcoholic Fatty Liver Disease	Recruiting

#### 3.2.3. Targeting Bile Acid Metabolism

Obeticholic acid (OCA) is a potent FXR agonist evaluated for the treatment of NASH-mediated fibrosis thanks to its ability to reduce liver fat and fibrosis in animal models of NAFLD. Currently being tested in phase III clinical trials [213], phase II studies demonstrated that OCA treatment improved multiple histological NASH features [214].

Aramchol is a fatty acid–BA conjugate that has demonstrated an ability to reduce liver fat and inflammation in NAFLD patients. Results from the phase IIb trial showed that aramchol was safe and well tolerated [215]. Although the primary end point of a reduction in liver fat did not meet the pre-specified significance level, the observed safety and changes in liver histology and enzymes encouraged the initiation of phase III trials [215], whose interim analysis revealed that the open-label part met its objectives (Table 3).

These treatments may be especially useful in the management of lean NAFLD as these patients have shown specific alterations of BA metabolism [55].

#### 3.2.4. Targeting Oxidative Stress, Inflammation and Fibrosis

N-acetylcysteine is frequently used where intracellular oxidant–antioxidant balance is concerned and it has protective effects against liver injury [216]. Its potential as antioxidant treatment in NAFLD has been demonstrated in animal models [217,218], whereas information in NAFLD patients is scarce but promising [216]. Thus, a phase III clinical trial evaluating the effect of N-acetylcysteine on markers of oxidative stress and insulin resistance in patients with NAFLD is currently recruiting participants (Table 3).

Pentoxifylline is a methylxanthine derivative with a variety of physiological effects at the cellular level, which include decreases in TNF-α gene transcription, affecting multiple steps in the cytokine/chemokine pathway that has been implicated in NAFLD pathogenesis. Thus, it has been evaluated in several clinical trials mostly showing beneficial effects in weight loss, improved liver function and histological changes in patients with NAFLD/NASH [219]. However, other studies have failed in demonstrating pentoxifylline’s effectiveness in reducing transaminases compared to placebos, and it did not positively affect any of the metabolic markers postulated to contribute to NASH [220].

Secukinumab is a monoclonal antibody against IL-17 used in the treatment of psoriasis. It has been shown to have neutral effects on fasting plasma glucose, lipid parameters and liver enzymes, while reducing levels of CRP, a marker for systemic inflammation, and markers of oxidative stress. Secukinumab produced improvements in arterial elasticity, coronary artery function and myocardial deformation indices, thus protecting from CVR [221]. However, publication of phase III clinical trial results evaluating liver function is pending (Table 3).

Finally, lubiprostone is a laxative drug that improves intestinal permeability. It was reported to ameliorate increases in intestinal permeability induced by a high-fat and high-cholesterol diet in an atherosclerosis mouse model, while in humans it improved the increased intestinal permeability induced by non-steroidal anti-inflammatory drugs. Thus, lubiprostone might prevent the excessive inflammation and fibrosis induced by gut-derived endotoxin in NAFLD patients [222]. Results from the phase IIa study have shown that lubiprostone was well tolerated and reduced the levels of liver enzymes in patients with NAFLD and constipation [222]. Therefore, recruitment for the phase III clinical trial is already open.

Treatments targeting inflammation and fibrosis might be eligible for patients with more advanced disease or those with enhanced inflammation due to co-morbidities such as IMIDs, whereas targeting intestinal permeability could be indicated for those with dysbiosis or IBD.

## 4. Concluding Remarks

The hallmark of NAFLD is the accumulation of lipids in the liver that results from deranged lipid metabolism. Consequently, NAFLD is strongly associated with obesity, insulin resistance and dyslipidemia. However, inflammation may precede steatosis as inflammatory events may lead to lipid accumulation. Therefore, there are many factors influencing NAFLD initiation and progression, such as environmental exposure, lifestyle, genetic susceptibility, metabolic status and the microbiome. The phenotypic manifestation of fatty liver diseases likely reflects the sum of the dynamic and complex systems-level interactions of these drivers; it follows that effective treatment requires that they be targeted with precision and based on a person’s phenotype [223]. Importantly, morbimortality in patients with NAFLD involves extra-hepatic organs as it is considered a mediator of systemic diseases including CVD. This further contributes to NAFLD’s heterogeneity, representing a major challenge in discovering highly effective therapies. Obtaining a comprehensive landscape of the main NAFLD drivers and patient outcome determinants should facilitate patient stratification and identification of disease subtypes with different natural history and liver disease courses. Therefore, a multi-omic and clinical data integration approach of NAFLD patients could help us to properly subphenotype and stratify patients, paving the way for precision medicine in NAFLD. On the other hand, implementing optimal strategies to promote physical activity, prescribing an appropriate diet and changing the model of care through the use of digital tools such as telemedicine are crucial elements that will help in maintaining healthy habits in patients with NAFLD, as well as being the current curative and preventive options. Prioritizing research in these areas and developing innovative strategies to address this growing public health concern is essential.

## Figures and Tables

**Table 1:** Genetic variants associated with NAFLD.

Gene	SNPs	Function	References
*PNPLA3*	rs738409	Lipid metabolism and inflammatory response	[70,71]
	rs6006460		
*MBOAT7*	rs641738	Lipid metabolism	[72]
*HSD17B13*	rs72613567	Lipid metabolism	[73]
*FTO*	rs1421085	Lipid metabolism and adipogenesis	[74]
*LPIN1*	rs13412852	Lipid metabolism and adipogenesis	[75]
*TM6SF2*	rs58542926	VLDL secretion	[76]
*LYPLAL1*	rs12137855	Glucose homeostasis	[77]
*GCKR*	rs780094	Regulation of de novo lipogenesis and insulin resistance	[78,79]
*ENPP1*	rs1044498	Insulin signaling inhibitor	[80]
*PPP1R3B*	rs4240624	Glycogen metabolism	[81]
*SOD2*	rs4880	Fibrosis and oxidative stress	[82]
*MERTK*	rs4374383	Immune response	[83]
*FNDC5*	rs3480	Liver fibrogenesis	[84]
*KLF6*	rs3750861	Liver fibrogenesis	[85]
*CDKN1A*	rs762623	Cell senescence	[86]
*IL28B*	rs12979860	Inflammatory response	[87]

**Table 2:** Steatogenic drugs.

Therapeutic Class	Drug/Group	References
Antiarrhythmics	Amiodarone	[126]
Antibiotics	Tetracyclines	[127,128]
Antidiabetics	Troglitazone	[129,130,131]
Antiepileptics	Carbamazepine	[132]
	Valproic acid	[132,133,134]
Anti-inflammatories	Dexamethasone	[135]
Antitumor drugs	5-Fluorouracil	[136,137]
	Irinotecan	[137,138,139,140]
	Leuprorelin acetate	[141]
	Methotrexate	[142]
	Tamoxifen	[143,144]
Antiretrovirals	Nucleoside Reverse Transcriptase Inhibitors	[145,146]
	Protease Inhibitors	[147]
Hormones	Estrogens	[148]
Vasodilator agents	Perhexiline maleate	[149,150]

## Data Availability

Not applicable.
